# Evaluation of mode of delivery in pregnant women infected with COVID-19

**DOI:** 10.18332/ejm/123888

**Published:** 2020-07-02

**Authors:** Paraskevi Giaxi, Elissavet Maniatelli, Victoria G. Vivilaki

**Affiliations:** 1 Midwifery Department, University of West Attica, Athens, Greece; 2. Second Department of Obstetrics and Gynecology, Aretaieion Hospital, National and Kapodistrian University of Athens, Athens, Greece

**Keywords:** COVID-19, pregnancy, mode of delivery, vaginal delivery section, midwifery-led models

Childbearing women and newborn infants continue to require safe family-centered care during the current COVID-19 pandemic and they represent a vulnerable population^1^. In the only published systematic review of 108 pregnancies infected with COVID-19, 50 women were delivered, 44 gave birth by cesarean section and only 6 women gave birth by vaginal delivery^2^.

The World Health Organization (WHO)^3^, Royal College of Obstetricians & Gynecologists and the Royal College of Midwives value equally each mode of delivery, however, opportunities should be provided to enable every woman to unlock their expectations and needs. Mode of birth should not be influenced by the presence of COVID-19, unless the woman’s respiratory condition demands urgent intervention for birth^3,4^. In a symptomatic woman who is becoming exhausted or hypoxic, an individualized informed choice should be made regarding the possibility of shortening the length of the second stage of labor with elective instrumental birth^4^.

Caesarean section is not a recommended method of childbirth in pregnant women infected with COVID-19, however this was the mode of delivery in the majority of cases^5,6^ with fetal distress cited as the indication behind the clinical decision. Moreover, ‘fetal distress’ is not justified by abnormal cardiotocograph findings or other factors like meconium stained amniotic fluid, abnormal foetal scalp lactate or blood flow changes. There was no evidence of COVID-19 in the amniotic fluid, umbilical cord blood, neonatal throat swab or breastmilk samples^5^. Vertical transmission was reported as negative in all neonates^7^.

Worldwide, CS rates are rising, and action needs to be taken, as was expressed in a recent Lancet series. CS rates in women with COVID-19 are even higher than in the general population. Protecting mothers from unnecessary medical technologies is one of the World Health Organization (WHO) strategies to promote maternal health. Under these circumstances, the role of midwife is more recognized as an advocate of natural birth for women^8^. Midwifery-led models of care are associated with lower cesarean utilization. The positive impact of midwife-led birth settings is well documented, including reductions in the need for a range of medical interventions^9,10^.These positive impacts remain of significant importance to prevent avoidable harm, and availability of midwife-led care settings for birth should therefore be continued as far as possible during the pandemic^1^.

The birth of COVID-19 pandemic is tokophobia. Frightened women are the products of this technocratic medicalized philosophy of birth. As ‘obedient’ consumers women are likely to choose intervention including Caesarean Section, distrusting the capacity of their own bodies to give birth normally. The influence of the media and significant technocratic believers, challenge the images of women and open the arena for a new understanding of safe birth during COVID-19 pandemic with Caesarean Section. Women need to be aware of their rights during this pandemic, that their own knowledge and feelings are valuable and, more importantly, that health research regarding COVID-19 is going on, however, Caesarean Section perinatal outcomes are well evident.
